# Cognition and Implementation of Disaster Preparedness among Japanese Dialysis Facilities

**DOI:** 10.1155/2021/6691350

**Published:** 2021-01-05

**Authors:** Hidehiro Sugisawa, Toshio Shinoda, Yumiko Shimizu, Tamaki Kumagai

**Affiliations:** ^1^Graduate School of Gerontology, J. F. Oberlin University, Machida-city, Tokyo 194-0294, Japan; ^2^Faculty of Health and Medical Sciences, Tsukuba International University, Tsuchiura-city, Ibaraki 300-0051, Japan; ^3^The Jikei University School of Nursing, Chofu-city, Tokyo 182-8570, Japan; ^4^School of Nursing, Osaka City University, Osaka-city 545-0051, Japan

## Abstract

**Introduction:**

Few quantitative studies have explored disaster preparedness in dialysis facilities worldwide. This study examined the levels of disaster preparedness and their related factors in dialysis facilities in Japan.

**Methods:**

We conducted a mail survey using a self-administered questionnaire for key persons responsible for disaster preparedness in dialysis facilities (*N* = 904) associated with the Japanese Association of Dialysis Physicians. Levels of disaster preparedness were evaluated by the implementation rates of four domains: (1) patient, (2) administration, (3) network, and (4) safety. Additionally, we focused on cognitive factors related to disaster preparedness, such as risk perception, outcome expectancy, self-efficacy, self-responsibility, and support from the surroundings.

**Results:**

A total of 517 participants answered the survey (response rate: 57.2%). Implementation rates differed according to the domains of disaster preparedness. While the average implementation rate of the safety domain was 81.8%, each average implementation rate was 57.9%, 48.3%, and 38.4% for the administration, network, and patient domains, respectively. The study found that self-efficacy and support from the surroundings of the participants were significantly associated with the four domains of disaster preparedness. Alternatively, risk perception and support from surroundings were significantly associated with one particular domain each.

**Conclusion:**

Our results suggest that boosting self-efficacy and support from surroundings among key persons of disaster preparedness in dialysis facilities may contribute to the advancement of the different domains of disaster preparedness.

## 1. Introduction

Natural disasters, such as earthquakes, hurricanes, and floods, can cause severe harm to patients undergoing hemodialysis. Even if dialysis facilities do not directly suffer material damage due to disasters, the disruption of infrastructure, such as water and power supply, and access to dialysis facilities prevents patients with end-stage renal diseases from receiving treatment [1]. As a result, patients needing hemodialysis indirectly face life crises even if they do not suffer any physical harm as a direct result of disasters. In fact, it was reported that 20% of patients who visited dialysis facilities in damaged areas missed three or more dialysis sessions due to Hurricane Katrina in the United States of America (USA) [2]. Missed dialysis sessions due to natural disasters such as earthquakes have also been reported in cases of other disasters, such as Hurricane Sandy [3], Hurricane Gustav [4], Chi-Chi earthquake [5], Marmara Earthquake [6], Hanshin-Awaji Earthquake [7], and the East Japan Earthquake [8].

To prevent and mitigate the suffering of hemodialysis patients due to natural disasters, disaster preparedness (DP) should be undertaken by all sectors: central and local governments, dialysis facilities, and dialysis patients [4]. In fact, there are already published manuals and guidebooks on DP for the use of dialysis facilities [9, 10] and hemodialysis patients [11, 12]. DP contributes to reducing missed dialysis sessions [4] and improving depressive symptoms and post-traumatic stress disorders (PTSD) among hemodialysis patients who have experienced a disaster [13]. Additionally, some studies have explored levels of DP and related factors to DP among patients receiving dialysis [14, 15]. However, only a few quantitative studies have focused on DP among dialysis facilities [16]. DP in medical facilities, especially hospitals, has been explored in several countries, such as the USA [17–20], Canada [21], Sweden [22], Switzerland [23], Japan [24–26], and China [27]. With this being said, with the exception of one study by Cliff et al. [28], there have only been a few studies on factors related to hospital DP. The research conducted by Cliff et al. examined the influences of risk perception among the chief executive officers of hospitals on DP. In addition, studies on cognitive factors related to DP among community residents have also been conducted. Previous research has even identified the consistent roles of cognitive factors such as self-efficacy, outcome expectancy, and personal responsibility in DP [28].

According to Becker et al. [29], there are three core cognitive factors related to DP: (1) hazard, (2) preparedness, and (3) personal cognition. First, hazard cognition is considered to be related to risk perception. Second, preparedness cognition describes one's understanding of the meaning of preparedness. Lastly, personal cognition expresses how people deal with a disaster. Therefore, this study explores factors related to the levels of DP among Japanese dialysis facilities by focusing on these three types of cognition from the perspective of individuals in charge of DP.

## 2. Methods

### 2.1. Participants

The potential participants were 904 individuals in charge of DP in the dialysis facilities under the Japanese Association of Dialysis Physicians—an organization composed of dialysis physicians, whose purpose is to conduct research, education, and training regarding dialysis and renal failure. At the end of 2018, there was a total of 4,458 dialysis facilities in Japan, of which approximately 20% were covered by this organization. As majority of dialysis facilities have a committee for DP, we sent out self-administrated questionnaires by mail in November 2019 to the possible participants in charge of DP in these facilities. If a dialysis facility did not have a DP committee, we sent out the questionnaires to the directors of these facilities instead. The deadline for responses was December 31, 2019. A total of 517 facilities participated in this survey, and the response rate was 57.2%.

### 2.2. Measurements

#### 2.2.1. Disaster Preparedness

**Figure 1:**
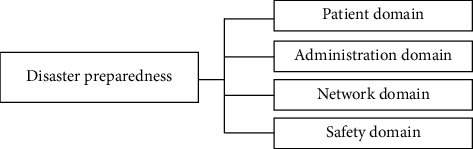
Domains of disaster preparedness.

Although some assessment scales have been created for DP in hospitals [30, 31], there were no assessment scales for DP in dialysis facilities. Therefore, we created original scales based on manuals on DP for dialysis facilities [9, 32, 33]. The newly created DP scales were separated into four domains: (1) patient, (2) administration, (3) network, and (3) safety as shown in Figure 1. Items included in each domain were reviewed by DP specialists from the Japanese Association of Dialysis Physicians to confirm content validity. Supplementary Table 1 shows all items included in each domain, and response categories for items, which were evaluated at the implementation of the item. We conducted categorical factor analyses to confirm whether the items in each domain could be treated as a one-dimensional scale. Categorical factor analyses were employed after the responses for each item were converted into binary data, which determined whether each item was included or not. Items with factor loadings under 0.32, as per convention [34], were deleted from each domain. In addition, the reliability of each domain was evaluated using Cronbach's alpha.

Ten items were used to evaluate the patient domain since the factor analyses indicated that factor loadings of all items on the first factor had values over the cut-off value. The reliability for this domain was 0.818. Eight items were used to evaluate the administration domain, although ten items were initially included. The two items that did not meet the cut-off value were deleted. The reliability was 0.750. The scale for the network domain included all five items subjected to factor analyses, and the reliability for this domain was 0.559. The scale for the safety domain was evaluated using four items, although this domain initially included six items. Similar to the administration domain, the two items that did not meet the cut-off point were deleted. The reliability for this domain was 0.399. Participants who completed at least 70% of the items in each scale were included in the analyses. Missing values were addressed by substituting these with the participants' mean for the completed scale items. Based on previous research, this method is appropriate for both random and non-random missing items in psychometric scale construction [35, 36].

#### 2.2.2. Cognitive Factors

As there are currently no scales designed to evaluate cognition of DP among persons in charge of DP, we again developed original scales. We evaluated the reliability of cognition scales just as we did for DP scales. Three cognitive factors (hazard beliefs, preparedness cognition, and personal beliefs) related to DP were measured using five separate scales: one scale on hazard beliefs (i.e., risk perception), one scale on preparedness cognition (i.e., outcome expectancy), and three scales on personal beliefs (i.e., self-efficacy, self-responsibility, and support from surroundings). We evaluated cognition of DP by hypothesizing the occurrence of a severe earthquake, such as the Hanshin-Awaji Earthquake. Questions evaluating cognition were answered with one of four choices: “Strongly Agree,” “Agree,” “Disagree,” and “Strongly Disagree.” Each choice was assigned a score from 4 to 1 for quantification. We evaluated the reliabilities of each cognition scale just as we did for the DP scales. Supplementary Table 2 shows all of the items divided according to cognition type.

Based on the scale used by Ejeta et al. [37], the scale for risk perception was created using questions that asked each perception of three disaster-related risks (i.e., occurrence of a largescale disaster in an area of the facility, large damage to the facility's buildings caused by a largescale disaster, and large damage to a lifeline in an area of the facility caused by a large scale disaster). Factor analyses supported that the three items were treated as a single dimension. The reliability for this scale was 0.495. The scoring of this scale was conducted by means of simple addition of the scores for each of the three items. The scoring of the four other scales (i.e., outcome expectancy, self-efficacy, self-responsibility, and support from surroundings) employed the same aforementioned method. The scale of outcome expectancy was also created by referring to the scale by Ejeta et al. [37]. We asked three questions regarding the evaluation of the effectiveness of current DP measures. Factor analyses indicated that this scale was composed of a single dimension and the reliability was 0.680. The scale for self-efficacy was created based on a scale by Yu et al. [38]. We asked whether the participants were confident in their ability to perform preparedness behaviors for three severe situations (i.e., when the facility has financial problems, when the staffs are very busy, and when the patients are unconcerned about a disaster). According to factor analyses, this scale was composed of a single dimension, with a reliability of 0.777. The scale for self-responsibility was developed based on a scale by Paek et al. [39]. Questions regarding the three types of self-responsibility were used to create the scale (i.e., progressing DP, saving patients' lives when an earthquake occurs, and saving staffs' lives when an earthquake occurs). Similar to the other scales, this was also composed of a single dimension based on the factor analyses. The reliability for this scale was 0.849. A scale for support from surroundings was created by using a scale by Story et al. [40]. It was composed of three questions about each of the three sectors (i.e., patients and their families, dialysis facilities in an area of the facility, and a local government in an area of the facility). It was treated as one dimension based on the result of the factor analyses and the reliability was 0.654.

#### 2.2.3. Control Factors

Characteristics of the participants and facilities which they worked for may influence the dialysis facilities' DP. We needed to control such influences to evaluate the independent influences of their cognitive factors on facilities' DP among the key persons. We assessed the jobs of the participants and their positions. Characteristics of the facilities included locations, types of establishing organization, and the number of patients undergoing dialysis. The location of the facility was divided into eight regional blocks, which is frequently used as an example of such a block in Japan. Based on the type of establishing organization, medical facilities are categorized as public or private and the size of the facility is decided based on the number of beds; hospitals have twenty or more beds, and clinics have nineteen or fewer beds. Based on the types of establishing organization and size, facilities were categorized as either public hospitals, private hospitals, or clinics. The number of dialysis patients was divided into quartiles.

### 2.3. Statistical Methods

The participants with no missing variables accounted for 91.3% of the data. Participants with one missing variable and two missing variables accounted for 4.1% and 2.9%, respectively. Participants with missing variables had significantly lower scores in the safety and network domains compared to those with no missing variables. Missing values were addressed by the full information maximum likelihood. We employed a multiple regression analysis by entering each scale of DP domains as a dependent variable and cognitive factors and control variables as independent variables. Data analyses were performed with Mplus ver. 7.4 software [41].

### 2.4. Ethical Considerations

This study was approved by the ethical review board of J. F. Oberlin University (approval number: 19028). The questionnaires were kept confidential. We regarded returned and completed questionnaires as an agreement to participate in this survey and we ensured participant privacy.

## 3. Results

### 3.1. Characteristics of the Participants and Their Facilities

As shown in Table 1, the participants were composed of dialysis physicians (47.8%), clinical engineers (24.2%), and nurses (17.6%). Regarding positions, the majority of participants were directors of facilities (33.1%), followed by heads of the dialysis department (31.5%), and nurses (12.8%). Participants' facilities included clinics (55.0%), public hospitals (15.1%), and private hospitals (29.9%). Most facilities were located in “Kanto” (22.2%), while the next most highly represented area was “Chub” (20.6%). The median of the total number of patients was 100.

### 3.2. Implementation Rates of Disaster Preparedness

Table 2 shows the implementation rates of disaster preparedness broken down by item and domains. Among the domains, the highest average implementation rate was 81.8% for the safety domain, and the range of rates of individual items was narrow (73.5% to 86.2%). The implementation rates for “Using a flexible tube in joint parts to fix the dialysis supply system and reverse osmosis membrane system on the wall” and “Fixing large medical equipment and putting them on an isolation device to prevent falling and shaking” were 86.2% and 73.5%, respectively. The second highest average implementation rate among the domains was the administration domain (57.9%). In this domain, the item with the highest implementation rate was “Disseminating information regarding how to handle withdrawal from dialysis at the time of disaster” (85.8%) and the range of rates of other items was 48.1–59.6%. The third highest implementation rate among the domains was network. Differences in implementation rates were large according to items: the highest rate was 75.2% for “Securing multiple means of communication” and the lowest rate was 30.1% for “Coming to an agreement regarding supply of medicines and equipment.” The lowest average implementation rate was 38.4% for the patient domain. Implementation rates differed according to the items. The rates ranged from 59.8% for “Securing the way of communication with their patients” to 14.8% for “Disseminating a contact address when people who cannot evacuate by themselves require evacuation.”

### 3.3. Cognitive Factors Related to Disaster Preparedness

As shown in Table 3, self-efficacy had a significant influence on all four dimensions. Support from the surroundings had significant influences on three dimensions, excluding the safety domain. Both risk perception and self-responsibility significantly influenced each dimension for the patient and the safety domains. Outcome expectancy significantly influenced the patient dimension, with a higher outcome expectancy leading to decreased levels of this dimension.

## 4. Discussion

Implementation rates differed by the domains of disaster preparedness. The study found that self-efficacy and support from the surroundings of the participants were significantly associated with three of the four domains of disaster preparedness. Alternatively, each cognition of risk perception, outcome expectancy, and self-responsibility was significantly associated with only a specific domain. Risk perception and outcome expectancy of the participants were significantly associated with the patient domain, and self-responsibility significantly influenced the safety domain.

According to a 2011 survey related to DP for all dialysis facilities in Japan [16], concerning the safety dimension, the rate of use of a flexible tube in joint parts to repair dialysis supply systems and reverse osmosis membrane systems was 52.9% (86.2% (rates in parentheses indicate rates for similar items in this study)), and the implementation rate of earthquake measures for reverse osmosis equipment and supply equipment was 48.4% (73.5%). Although we deleted two items—“Lock bed casters” and “Earthquake measures for console”—in order to develop the scale as a single dimension, the implementation rates of these two items rates in the 2011 survey were 93.2% (95.5%) and 92.3% (94.8%), respectively. These results suggest that the implementation rates of items regarding the safety domain are relatively high and the rates of some items have improved by 20–30% over the last 10 years. This study also added items regarding the general safety domain in medical facilities (i.e., “Checking the buildings in the facility from the perspective of disaster preparedness”). The implementation rates of these items were over 80%, suggesting that most dialysis facilities in Japan put the general safety domain into practice. As hospital and clinic buildings are regulated by several laws, such as the Building Standards Law, Fire Prevention Law, and Medical Care Law [26], these laws may have contributed to the improvement of the safety domain.

The items regarding the administration domain were not specific to dialysis facilities, with the exception of one item (i.e., “Disseminating information regarding how to handle withdrawal from dialysis at the time of disaster”). The implementation rate of this dialysis-specific item was 85.8%, which was very close to the rate of 90.4% in the 2011 survey. Other items were also important for general medical facilities, including dialysis facilities. Our results indicate that the average implementation rate of these items was 57.9%, which is lower than the rate for the general safety domain. For the network domain, we added the following item: “Discussing securing power and water supply at the time of disaster with related organizations.” Large hospitals are required to prepare a supply of water and power during disasters of their own accord. However, it is unrealistic for clinics to do so. Hence these facilities require support from regional governments and power companies for their water and power supply. The average implementation rate of the network domain was under 50%. In addition, we inquired about what types of information the facilities provided to the patients. Rates of information provision by the facilities varied by a wide percentage among different types: the minimum rate was 14.8%, maximum rate was 59.8%, and the average implementation rate of the patient domain was under 40%. This suggests that the administration, network, and patient domains have much room for improvement in their implementation rates.

This study also aimed to explore the factors related to DP, focusing on cognitive factors in particular. As it is possible that the participants' job types and their positions in their respective facilities had influences on DP and could have confounded the influences of cognition on DP, we controlled for these variables by adding them to our analytical models. As a result, self-efficacy was significantly associated with the progress of all four domains even after controlling for these variables. In addition, other than the safety domain, support from the surroundings was significantly associated with three of the domains. This suggests that boosting self-efficacy and support from surroundings among key persons in charge of DP is important for improving overall levels of DP domains. The study also found that risk perception and self-responsibility were significantly associated with a specific domain: risk perception with the patient domain and self-responsibility with the safety domain. In terms of risk perception, Cliff et al. [28] did not observe significant differences in the overall DP between high and low levels of risk perception. According to a study by Samaddar et al. [42], flood risk perception had little to no correlation with household food preparedness. With this being said, our study provides evidence that shows that risk perception influences DP in dialysis facilities. It is hypothesized that self-responsibility increases disaster preparedness among residents in a community [43]. However, studies examining these relationships provide inconsistent results [44, 45]. In our study, this hypothesis was partially supported. Accordingly, programs focusing on boosting risk perception and self-responsibility among key persons may assist in improving the patient and safety domains in dialysis facilities.

Interestingly, outcome expectancy had a significant negative influence on the patient domain, contrary to our expectations, and no significant influences on the other three domains. In the analyses of bivariate relationships, outcome expectation had a positive, although non-significant, association with the patient domain. When two types of cognitive factors, self-efficacy and support from surroundings, were added in the regression model, outcome expectancy had a significant negative influence on the patient domain. While outcome expectancy had a strong correlation with self-efficacy (*r* = 0.527) and social support from surroundings (*r* = 0.525), there was no multicollinearity between outcome expectancy and both variables. Although we evaluated cognition regarding whether or not DP contributes to “maintaining the provision of dialysis” and “saving patients' lives” to evaluate levels of outcome expectancy, we did not include cognition regarding the possibility of enrichment of the patient domain due to DP. Under these restrictions, the participants may have answered questions regarding outcome expectancy based on the assumption that these entailed only the “safety” and “administration” domains of DP. Accordingly, it can be inferred from our results that the key persons with higher outcome expectancies, which was specific to the safety and administration domains, are more likely to downplay the importance of the patient domain. Either way, the validity of this finding requires further examination.

This study has several limitations. The first is that the generalizability of these findings should be undertaken with caution, given the relatively low response rate and limited number of participants. Non-participants in this study, which accounted for 40% of the total participants, may not only be uninterested in DP but also have lower implementation rates of DP in their facilities than the actual participants. In addition, the organization that cooperated with us on this study is able to disseminate DP information to its members. The second limitation is associated with the validity of the created scales. This study used self-report questionnaires for the sake of efficiency to gather information on the levels of DP from many facilities. However, social desirability can lead to response bias in self-report research [46]. Due to social desirability, participants may have answered questions regarding DP in a manner that they feel is more socially acceptable rather than being completely truthful. Considering the possibility that these first two limitations may have induced bias in our findings, DP among dialysis facilities may be worse than our results indicate. The third limitation is related to the low reliability of some domains of DP, especially the safety domain. Clearly, there is a need to create a highly reliable scale. Although two cognitive factors had a significant influence on the safety domain in this study, the scales with lower reliability had a weaker influence on dependent variables in general. The fourth limitation is that we did not address other factors related to DP, such as economic constraints. A study on hospital DP by Ochi et al. indicated that hospitals that provided chronic care were less likely to have sufficient stockpiles of supplies [26]. They suggest that some hospitals were more likely to be under economic constraints and thus attempt to reduce inventory costs. Recent revisions of medical fees, such as decreases in outpatient medical management fees for hemodialysis patients and package pricing, have induced severe financial crises among dialysis facilities [47]. Time and personnel allocated for DP, especially the administration domain, may appear wasteful from a shortsighted point of view, as disasters are not always imminent. As a result, financial constraints may prevent the advancement of DP.

## 5. Conclusions

This study examined the levels of disaster preparedness and the other related factors in dialysis facilities in Japan. We focused on cognitive factors related to disaster preparedness, such as risk perception, outcome expectancy, self-efficacy, self-responsibility, and support from the surroundings. The results showed that self-efficacy was significantly associated with the progress of all four domains even after controlling for other three cognitive factors. In addition, support from the surroundings was significantly associated with the three domains. Alternatively, each cognition of risk perception, outcome expectancy, and self-responsibility was significantly associated with only a specific domain. Our results suggest that boosting self-efficacy and support from surroundings among key persons of disaster preparedness in the dialysis facilities may contribute to the advancement of the overall levels of DP domains.

## Figures and Tables

**Table 1:** Characteristics of participants and their facilities.

Variables	Percentage of missing values	Categories/characteristics	Distribution
*Participants*			
Job type	0.0^1)^	Physician	47.8
		Nurse	17.6
		Clinical engineer	24.2
		Clerical staff	8.1
		Others/nonresponse	2.3
Position	0.0^1)^	Director of the facility	33.1
		Office manager	7.9
		Head of the dialysis department	31.5
		Chief nurse	12.8
		Chief clinical engineer	7.4
		Others/nonresponse	7.4
*Cognitive factors*			
Hazard belief	0.2	Mean (SD)	9.15 (1.53)
Outcome expectancy	1.5	Mean (SD)	9.09 (1.65)
Self-efficacy	1.4	Mean (SD)	7.63 (1.67)
Self-responsibility	0.8	Mean (SD)	11.0 (1.34)
Support from surroundings	2.1	Mean (SD)	8.67 (1.71)

*Facility characteristics*			
Location of the facilities by the eight regional divisions	0.6	Hokkaido	4.9
		Tohoku	6.8
		Kanto	22.2
		Chubu	20.6
		Kansai	15.4
		Chugoku	7.0
		Shikoku	5.6
		Kyushu	17.5
Founder and size	1.2	Public hospitals	15.1
		Private hospitals	29.9
		Clinics	55.0
Number of total dialysis patients	1.4	First quartile: 60 or less patients	27.3
		Second quartile: 61–100	24.1
		Third quartile: 101–160	24.7
		Fourth quartile: above 160	23.9

^1)^As cases with missing values were added under the “Others” category, the percentage of missing values was 0.

**Table 2:** Implementation rates of disaster preparedness broken down by item and domains.

Items regarding disaster preparedness	Implementation rate^1^	Rate of missing values
*Patient*		
Supervising check dry weight	48.9	0.0
Disseminating means of communication to the facility at the time of disaster	56.9	0.0
Disseminating dietary management at the time when dialysis interval is longer	31.2	1.9
Disseminating a contact address when people who cannot evacuate by themselves want to evacuate	14.8	1.9
Disseminating information regarding how to check whether the facility is open or not at the time a disaster	41.7	2.1
Disseminating a backup system when the facility does not work due to a disaster	19.6	2.1
Disseminating the way patients behave when a disaster happens during a dialysis session	41.9	2.1
Disseminating procedures, routes, and places of evacuation in the facility	32.4	2.1
Encouraging the identification of an evacuation place in advance	36.0	2.1
Securing the way of communication with their patients	59.8	0.0
Average implementation rates of items in the patient domain	38.4	2.1

*Administration*		
Establishing a committee of disaster preparedness and discussing regularly	50.6	0.2
Making a manual for disaster preparedness and revising it	57.3	0.0
Assigning roles to staff at the time of a disaster	52.8	2.1
Conducting annual disaster drills for the staff and ensuring the assignment of roles	59.6	2.3
Disseminating information regarding how to handle withdrawal from dialysis at the time of disaster	85.8	2.1
Checking regularly whether the contact network system among the staff is working	48.1	0.2
Preparing an emergency kit and being ready to use it	54.5	2.1
Keeping goods for disaster preparedness in an accessible place for the staff	53.2	2.1
Average implementation rate of items in the administration domain	57.9	2.3

*Network*		
Discussing securing power and water supply at the time of disaster with related organizations	47.8	0.4
Securing multiple means of communication at the time of a disaster	75.2	0.2
Concluding an agreement about the supply of medicines and equipment at the time of disaster with related organizations	30.1	0.4
Concluding an agreement about mutual help at the time of a disaster with other facilities	55.6	0.8
Understanding levels of emergency supplies of power and water at the time of a disaster from related organizations	31.9	0.6
Average implementation rate of items in the networks domain	48.3	0.8

*Safety*		
Using a flexible tube in joint parts to fix the dialysis supply system and reverse osmosis membrane system on wall	86.2	0.4
Checking the buildings in the facility from points of view of disaster preparedness	84.1	0.2
Fall prevention of items and stopping the placement of seats and beds under places with potential dangers of falling objects	83.5	0.6
Fixing large medical equipment and putting them on an isolation device to prevent falling and shaking	73.5	0.8
Average implementation rate of items in the safety domain	81.8	0.8

^1^Implementation rates were calculated after excluding cases with missing values for each item. Please refer to the Methods section for further details.

**Table 3:** Factors related to implementation rates of disaster preparedness divided by domains using standardized coefficients.

Variables		Patients	Administration	Networks	Safety
Cognitive factors	Risk perception	0.098^*∗*^ [0.015, 0.182]	0.080 [−0.003, 0.162]	0.005 [−0.078, 0.089]	−0.046 [−0.127, 0.034]
	Outcome expectancy	−0.088^*∗*^ [−0.174, −0.001]	−0.061 [−0.147, 0.025]	0.087 [−0.004, 0.179]	−0.077 [−0.176, 0.023]
	Self-efficacy	0.236^*∗∗∗*^ [0.142, 0.331]	0.209^*∗∗∗*^ [0.118, 0.300]	0.115^*∗*^ [0.020, 0.0.210]	0.269^*∗∗∗*^ [0.172, 0.367]
	Self-responsibility	0.006 [−0.082, 0.094]	0.065 [−0.029, 0.160]	−0.024 [−0.114, 0.067]	0.098^*∗*^ [0.006, 0.191]
	Support from surroundings	0.213^*∗∗∗*^ [0.119, 0.307]	0.174^*∗∗∗*^ [0.079, 0.269]	0.152^*∗∗*^ [0.065, 0.239]	0.030 [−0.064, 0.124]
Job types of participants	Physician	Reference	Reference	Reference	Reference
	Nurse	0.086 [−0.46, 0.217]	0.105 [−0.056, 0.266]	−0.021 [−0.150, 0.109]	0.091 [−0.093, 0.276]
	Clinical engineer	0.025 [−0.107, 0.157]	−0.044 [−0.187, 0.098]	0.009 [−0.132, 0.149]	−0.071 [−0.211, 0.070]
	Clerical staff	0.239*∗* [0.045, 0.433]	−0.080 [−0.341, 0.181]	0.010 [−0.184, 0.203]	0.190 [−0.083, 0.463]
	Others/nonresponse	−0.003 [−0.102, 0.096]	−0.055 [−0.147, 0.037]	0.076 [−0.030, 0.182]	0.012 [−0.053, 0.073]
Position of participants	Director of the facility	Reference	Reference	Reference	Reference
	Office manager	−0.205*∗* [−0.401, −0.010]	−0.002 [−0.269, 0.265]	−0.078 [−0.271, 0.115]	−0.232 [−0.512, 0.043]
	Head of dialysis department	−0.127 [−0.268, 0.014]	−0.072 [−0.209, 0.065]	−0.109 [−0.247, 0.030]	−0.128 [−0.266, 0.011]
	Chief nurse	−0.021 [−0.164, 0.122]	−0.087 [−0.245, 0.072]	−0.016 [−0.152, 0.120]	−0.194^*∗*^ [−0.380,−0.008]
	Chief clinical engineer	−0.063 [−0.177, 0.051]	0.018 [−0.098, 0.134]	−0.013 [−0.136, 0.111]	−0.031 [−0.149, 0.088]
	Others/nonresponse	−0.087 [−0.205, 0.031]	0.048 [−0.081, 0.178]	−0.049 [−0.152, 0.054]	−0.024 [−0.137, 0.088]
Location of the facilities	Hokkaido	−0.085 [−0.178, 0.007]	−0.076 [−0.167, 0.105]	−0.038 [−0.131, 0.056]	−0.084 [−0.184, 0.016]
	Tohoku	−0.076 [−0.170, 0.017]	−0.018 [−0.099, 0.062]	0.102^*∗*^ [0.017, 0.188]	−0.041 [−0.126, 0.044]
	Kanto	Reference	Reference	Reference	Reference
	Chubu	0.029 [−0.073, 0.131]	0.048 [−0.053, 0.149]	0.068 [−0.027, 0.164]	−0.046 [−0.148, 0.056]
	Kansai	0.007 [−0.089, 0.103]	−0.050 [−0.146, 0.046]	−0.119^*∗*^ [−0.214, −0.024]	−0.037 [−0.133, 0.058]
	Chugoku	0.003 [−0.082, 0.088]	−0.018 [−0.106, 0.070]	0.061 [−0.043, 0.165]	−0.078 [−0.168, 0.012]
	Shikoku	0.014 [−0.064, 0.093]	−0.005 [−0.073, 0.062]	0.018 [−0.066, 0.102]	0.030 [−0.047, 0.108]
	Kyushu	−0.059 [−0.150, 0.032]	−0.060 [−0.158, 0.038]	−0.063 [−0.160, 0.033]	−0.112^*∗*^ [−0.207, −0.016]
Founder and size	Public hospitals	0.012 [−0.092, 0.117]	0.179^*∗∗*^ [0.079, 0.290]	0.251^*∗∗∗*^[0.145, 0.356]	0.143^*∗∗*^ [0.049, 0.237]
	Private hospitals	−0.136^*∗∗*^ [−0.230, −0.043]	−0.025 [−0.117, 0.067]	0.006 [−0.086, 0.099]	0.017 [−0.080, 0.113]
	Clinics	Reference	Reference	Reference	Reference
The number of total dialysis patients	First quartile: 60 or less patients	Reference	Reference	Reference	Reference
	Second quartile: 61–100	0.068 [−0.030, 0.166]	0.132^*∗∗*^ [0.037, 0.226]	0.033 [−0.064, 0.130]	0.039 [−0.066, 0.144]
	Third quartile: 101–160	0.101 [−0.002, 0.204]	0.089 [−0.017, 0.195]	0.065 [−0.037, 0.166]	0.009 [−0.096, 0.114]
	Fourth quartile: above 160	0.094^*∗*^ [0.002, 0.187]	0.223^*∗∗∗*^ [0.125, 0.322]	0.110^*∗*^ [0.009, 0.212]	0.091 [−0.010, 0.193]
*R* square		0.239^*∗∗∗*^	0.229^*∗∗∗*^	0.229^*∗∗∗*^	0.161^*∗∗∗*^

^*∗*^ *P* < 0.05, ^*∗∗*^*P* < 0.01, and ^*∗∗∗*^*P* < 0.001. : confidence interval.

## Data Availability

The data are available from the corresponding author upon reasonable request.

## Supplementary Materials

Supplementary Materials Supplementary Table 1: items in four domain and response categories classified by implementation/non-implementation. Supplementary Table 2: items in scales of each cognition.
