# Supplementary material for: Cognition and Implementation of Disaster Preparedness among Japanese Dialysis Facilities
Source: Int J Nephrol. 2021 Jan 5;2021:6691350. doi: 10.1155/2021/6691350 (PMC7803413; doi:10.1155/2021/6691350)
Supplement: Supplementary Materials — Supplementary Table 1: items in four domain and response categories classified by implementation/non-implementation. Supplementary Table 2: items in scales of each cognition. [file 6691350.f1.zip › 6691350.f1/Supplementary_Table_1_final (1).docx]

**Supplementary table 1: Items in four domain and response categories classified by implementation/non implementation**

| **Items regarding disaster preparedness**^1)^ | **Response categories** | |
| --- | --- | --- |
|  | **Implementation** | **Non-**  **Implementation** |
| 【Patient】 |  |  |
| *Do you supervise dry weight checks? | Regularly supervise | Irregularly supervise/  Don’t supervise |
| *Do you disseminate means of communication to the facility at the time of disaster? | Do so both in writing and verbally | Do them in writing or verbally/ Don’t do them/ Don’t have means of communication |
| *Do you disseminate dietary management at the time when dialysis interval is longer? | Do so both in writing and verbally | Do them in writing or verbally/ Don’t do them |
| *Do you disseminate a contact address when people who can’t evacuate by themselves want to evaluate? | Do so both in writing and verbally | Do them in writing or verbally/ Don’t do them |
| *Do you disseminate how to check whether the facility open or not at the time a disaster? | Do so both in writing and verbally | Do them in writing or verbally/ Don’t do them |
| *Do you disseminate backup system when the facility doesn’t work by a disaster? | Do so both in writing and verbally | Do them in writing or verbally/ Don’t do them |
| *Do you disseminate the way patients behave when a disaster happens during taking dialysis? | Do so both in writing and verbally | Do them in writing or verbally/ Don’t do them |
| *Do you disseminate procedures, routes, and place of evacuation in the facility? | Do so both in writing and verbally | Do them in writing or verbally/ Don’t do them |
| *Do you Encourage to identify evacuation place in advance? | Do so for each patient | Do through patient group/ Don’t do it |
| *Do you secure the way of communication with your patients? | Do so | Don’t do it |
| 【Administration】 |  |  |
| *Do you establish committee of disaster preparedness and discuss regularly? | Do so regularly | Establish it, but don’t have the meeting regularly/don’t establish it |
| *Do you make a manual for disaster preparedness and revise it regularly? | Make and revise it regularly | Make but don’t revise it regularly |
| *Do you assign roles for staffs at the time of a disaster? | Decide rolls of all staffs | Decide rolls of a part of staffs/don’t decide |
| *Do you conduct disaster drills by the staff annually and make sure of assignments of roles? | Do them annually | Conduct them annually, but don’t make sures of roles annually/Don’t do them annually |
| *Do you disseminate how to withdrawal from dialysis at the time of disaster? | Do so | Don’t do it |
| *Do you check regularly whether contact network system among staffs works or not? | Do so regularly | Don’t do regularly/Don’t have contact network |
| Do you apply non-regulated vehicles to transport their patients at the time of disaster? | Do so | Don’t do it |
| *Do you prepare an emergency kit and are you ready to bring it soon? | Do so | Do a part of it/ Don’t do it |
| *Dou you keep goods for disaster preparedness in the place the staffs can take out right way? | Do so | Do a part of it/ Don’t do it |
| Do you stock equipment and medicine for dialysis which are necessary to sustain dialysis during over three days? | Do them | Don’t do them |

**Supplementary table 1 (continued)**

| 【Networks】 |  |  |
| --- | --- | --- |
| *Do you discuss securing power and water supply at the time of disaster with related organizations? | Do this through area level or prefecture level organizations in which dialysis facilities participate/Do so individually | Don’t do it |
| *Do you secure multiple means of communication at the time of a disaster? | Do so | Don’t do them |
| *Do you conclude an agreement about supply of medicines and equipment at the time of disaster with related organizations | Do so | Don’t do it |
| *Do you conclude an agreement about mutual help at the time of a disaster with other facilities? | Do so | Don’t do it |
| *Dou you understand levels of emergency supply of power and water at the time of a disaster from related organizations? | Do so | Don’t do it |
| 【Safety】 |  |  |
| Do you unlock casters of patient monitoring device? | Do so | Don’t do it |
| Dou you lock casters of patients’ beds. | Do so | Don’t do it |
| *Do you use a flexible tube in joint parts to fix dialysis supply system and reverse osmosis membrane system on wall? | Do so | Don’t do it |
| *Do you check the buildings in the facility from points of view of disaster preparedness? | Do so | Don’t do it |
| *Do you take measures of fall prevention of items of TV and stop putting seats and beds under places with dangers due to falling objects? | Do so | Don’t do it |
| *Do you fix large medical equipment and put large them on an isolation device to prevent fall and shaking them? | Do so | Don’t do it |

Note1: ＊means items that were used to create scales of each domain.
