# Supplementary material for: Cognition and Implementation of Disaster Preparedness among Japanese Dialysis Facilities
Source: Int J Nephrol. 2021 Jan 5;2021:6691350. doi: 10.1155/2021/6691350 (PMC7803413; doi:10.1155/2021/6691350)
Supplement: Supplementary Materials — Supplementary Table 1: items in four domain and response categories classified by implementation/non-implementation. Supplementary Table 2: items in scales of each cognition. [file 6691350.f1.zip › 6691350.f1/Supplementary_Table_2_final (1).docx]

**Supplementary Table 2: Items in scales of each Cognition ^1)^**

| 【Risk perception】 |
| --- |
| Dou you think that a massive earthquake will happen in this area within five years? |
| Do you think that buildings of this facility will suffer serious damage when a massive earthquake hits this area? |
| Do you think that it is highly probable that power and water supplies will be disrupted when such a massive earthquake occurs in this area? |
| 【Outcome expectancy】 |
| Do you think that preparation for an earth quake can minimize influence on medical care provided by the facility? |
| Do you think that it can save patients’ lives who use your facility? |
| Do you think preparation for an earth quake can enhance the reputation of your facility? |
| 【Self-efficacy】 |
| Do you have confidence in advancing disaster preparedness even when you the respondent’s facility faces financial problems? |
| Do you have confidence in advancing disaster preparedness even when staff members who work for the respondent’s facility are very busy? |
| Do you have confidence in advancing disaster preparedness even when patients who use the respondent’s facility are unconcerned about an earthquake? |
| 【Responsibility】 |
| Do you think that you have a responsibility for progressing earthquake preparedness? |
| Do you think that you have a responsibility for saving patients’ lives when an earthquake occurs? |
| Do you think that you have a responsibility for saving staffs’ lives when an earthquake occurs? |
| 【Support from surroundings】 |
| Do you think that your patients and their families are proactive in preparedness for an earthquake disaster? |
| Do you think that dialysis facilities in this area are proactive in preparation for an earthquake disaster? |
| Do you think that a local government located in this area are proactive in preparation for an earthquake disaster? |

Note1: Choices to answer for all questions were four points: “strongly agree, “agree”, “disagree” and “strongly disagree”.
